# Parameter identifiability in evidence-accumulation models: The effect of error rates on the diffusion decision model and the linear ballistic accumulator

**DOI:** 10.3758/s13423-024-02621-1

**Published:** 2025-01-07

**Authors:** Malte Lüken, Andrew Heathcote, Julia M. Haaf, Dora Matzke

**Affiliations:** 1 Netherlands eScience Center, Science Park 402, 1098 XH Amsterdam, The Netherlands; 2 Department of Psychology, University of Amsterdam, Amsterdam, The Netherlands; 3 Department of Psychology, University of Potsdam, Potsdam, Germany

**Keywords:** Decision making, Response times, Parameter recovery, Bayesian statistics, Esxperimental design

## Abstract

A variety of different evidence-accumulation models (EAMs) account for common response time and accuracy patterns in two-alternative forced choice tasks by assuming that subjects collect and sum information from their environment until a response threshold is reached. Estimates of model parameters mapped to components of this decision process can be used to explain the causes of observed behavior. However, such explanations are only meaningful when parameters can be identified, that is, when their values can be uniquely estimated from data generated by the model. Prior studies suggest that parameter identifiability is poor when error rates are low but have not systematically compared this issue across different EAMs. We conducted a simulation study investigating the identifiability and estimation properties of model parameters at low error rates in the two most popular EAMs: The diffusion decision model (DDM) and the linear ballistic accumulator (LBA). We found poor identifiability at low error rates for both models but less so for the DDM and for a larger number of trials. The DDM also showed better identifiability than the LBA at low trial numbers for a design with a manipulation of response caution. Based on our results, we recommend tasks with error rates between 15% and 35% for small, and between 5% and 35% for large trial numbers. We explain the identifiability problem in terms of trade-offs caused by correlations between decision-threshold and accumulation-rate parameters and discuss why the models differ in terms of their estimation properties.

In psychological experiments, speeded decisions are commonly described by response times (RTs) and accuracy. For instance, in the classic lexical decision task (Meyer & Schvaneveldt, 1971), participants press a left or right button depending on whether stimuli are words or nonwords. RT is typically measured as the time between stimulus onset and button press and accuracy as the proportion of correct responses. RT and accuracy patterns observed in these two-alternative forced choice (2AFC) tasks are governed by experimental manipulations. For example, when a task becomes more difficult, subjects tend to respond more slowly and less accurately.

Joint RT and accuracy patterns in 2AFC tasks can be accounted for by evidence-accumulation models (EAM; see Donkin and Brown, 2018, for a review). EAMs assume that decision-makers sequentially accumulate (noisy) information from their environment that favors one of the available responses. When the amount of information regarding one choice reaches a threshold, accumulation stops, and the decision-maker executes the corresponding response.

**Fig. 1:**
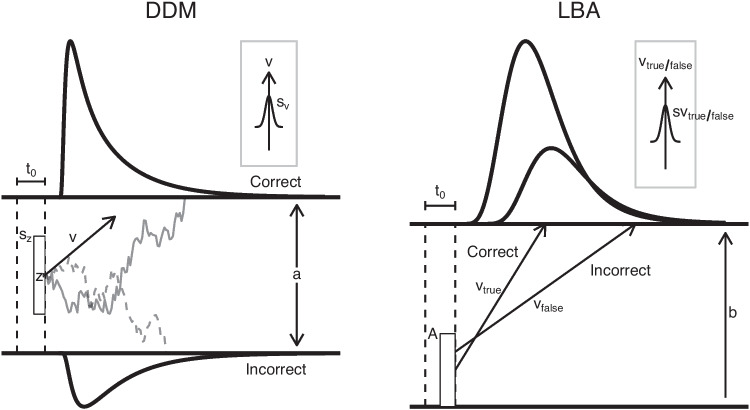
Graphical illustration of the diffusion decision model and the linear ballistic accumulator. Diffusion decision model (DDM): Evidence accumulation starts at *z* and drifts towards the upper (*solid trajectory*) or lower response threshold (*dashed trajectory*). The thresholds are separated by *a*. Starting points vary across trials with uniform range $s_z$ and drift rates according to a normal distribution with mean *v* and standard deviation $s_v$. Linear ballistic accumulator (LBA): Two independent accumulators start at uniformly distributed starting points with range *A* and grow linearly until one of them reaches the decision threshold *b*. The accumulation rates are normally distributed across trials with mean $v_{\text {true}}$ and standard deviation $s_{v_\text {true}}$ for the accumulator for the response that matches the stimulus, and $v_{\text {false}}$ and $s_{v_\text {false}}$ for the accumulator that mismatches the stimulus. In both models, the duration of processes that are not part of the decision-making process (e.g., encoding and motor execution) are captured by the non-decision time parameter $t_0$. Densities represent the response time distributions for correct and incorrect responses

Whereas traditional analyses treat RTs and accuracy separately, EAMs simultaneously account for both measures and capture multivariate effects (Voss et al., 2013). As computational models, they formalize the theory of sequential sampling and allow researchers to make precise, falsifiable predictions about decision making in 2AFC tasks. Moreover, EAMs are suitable measurement tools that quantify individual differences in the cognitive processes represented by the model parameters (Donkin & Brown, 2018; Forstmann et al., 2016; van der Maas et al., 2011). Thus, by decomposing observed performance in terms of the underlying cognitive processes and separating different sources of variability, EAMs can enhance the specificity and sensitivity of inferences drawn from choice RT data. The most popular EAMs are the diffusion decision model (DDM; Ratcliff, 1978, Ratcliff and McKoon, 2008) and the linear ballistic accumulator (LBA; Brown and Heathcote, 2008), both illustrated in Fig. 1.

The DDM describes evidence accumulation as a single fractional Gaussian drift process that is bounded by an upper and lower threshold. The process has a starting point denoted by *z* and drifts with rate *v* and standard deviation *s*. The response thresholds are separated by the distance *a*. Accumulation stops when the process reaches the upper or lower threshold. The time between the beginning and end of the accumulation process is the decision time $t_d$. Processes that are not part of the decision-making process (e.g., encoding the stimuli and executing a motor response) are captured in the non-decision time parameter $t_0$. Observed RT is given by the sum of $t_d$ and $t_0$ (Ratcliff, 1978; Ratcliff & McKoon, 2008; Voss et al., 2013; Stone, 1960). The DDM has been extended several times to more accurately account for typical RT patterns (Ratcliff et al., 2016). To accommodate different RT distributions for correct and error responses, Ratcliff (1978) allowed drift rate to vary across trials according to a normal distribution with mean *v* and standard deviation $s_v$. Furthermore, Ratcliff and Rouder (1998) added variability in starting point *z*, allowing it to vary across trials with range $s_z$ (see also Laming, 1968). Lastly, Ratcliff and Tuerlinckx (2002) added a uniform $t_0$-variability with range $s_{t_0}$.

In contrast to the DDM, the LBA assumes independent, linear accumulation processes for each response that are only bounded by an upper threshold. Each accumulator starts at a uniformly distributed value with range *A* and increases linearly towards the threshold. The slopes of the accumulator trajectories (drift rates) vary across trials according to Gaussian distributions with different means and standard deviations for the accumulator for the response that matches the stimulus ($v_{\text {true}}$ and $s_{v_\text {true}}$) and mismatches the stimulus ($v_{\text {false}}$ and $s_{v_\text {false}}$). The decision process stops when the first accumulator reaches its threshold *b*; the response is determined by the ”winning” accumulator. Starting point variability *A* and decision threshold *b* can vary between accumulators. The LBA also includes a non-decision time parameter $t_0$ and assumes that observed RT is the sum of decision and non-decision time (Brown & Heathcote, 2008).

An attractive characteristic of EAMs is that their parameters can be used to quantify the different components of the decision process (Turner et al., 2017). However, parameter estimates can only be meaningfully interpreted when they are unique for a given dataset (Moran, 2016; van Maanen & Miletić, 2020). This property of *parameter identifiability* depends both on the experimental design from which data are obtained (Turner et al., 2013; Spektor & Kellen, 2018) and the mathematical structure of the model^1^. For most EAMs, one or more parameters must be fixed to ensure identifiability (Donkin et al., 2009), conventionally $s = 1$ in the DDM and $s_{v_\text {false}}=1$ in the LBA. However, even then, identifiability can remain problematic (e.g., Evans, 2020; Ratcliff and Tuerlinckx, 2002; Boehm et al., 2018; Visser and Poessé, 2017; Turner et al., 2013; Palestro et al., 2018) because EAM parameters are often “sloppy” (Brown & Sethna, 2003), that is, highly correlated. If two parameters are highly correlated, they can trade off without much changing the predictions of the model. Thus, for a given dataset, there are multiple sets of parameter estimates that yield almost equivalent model fit, making parameter-based inference ambiguous.

Previous studies suggest that error responses play an important role in parameter identifiability. Ratcliff and Tuerlinckx (2002) explain parameter identifiability issues in the DDM by extremely slow error responses that shift parameters in the same direction away from their true values, leading to correlations among them (p. 454). Ratcliff (2014) examined estimation performance for the DDM in 11 datasets from different perceptual discrimination tasks with a range of difficulty conditions causing error rates to vary from near floor to ceiling. The difficulty manipulation was entirely accounted for by the accumulation rate parameters, with the other parameters held constant across conditions. Consistent with good identifiability, the rate estimates decreased as difficulty increased, leading to the conclusion that accumulation rates can be identified even when error rates are low, as long as enough errors occur in other conditions and other parameters are fixed across these conditions. Ratcliff (2014), however, did not give recommendations on how many errors are necessary to identify parameters in a single condition. For the LBA, Heathcote et al. (2019) used simulations to show better estimation performance when the data included 25% compared to 2.5% errors but again did not make any specific recommendations.

Although demonstrating the importance of errors, previous studies do not provide a clear picture of how error rates influence parameter identifiability in the DDM and LBA. This study aims to fill this gap by systematically manipulating the prevalence of errors in simulated data and analyzing how correlations between parameter estimates affect parameter identifiability at different levels of error rates. Our analysis may provide caveats for the interpretation of past studies that had applied the DDM and LBA to datasets with extremely low error rates (e.g., O’Callaghan et al., 2017; Hartmann et al., 2019; Mattes et al., 2020), potentially questioning the reliability and validity of the reported parameter estimates and the inferences based on them.

## Method

We conducted three studies simulating binary choices and corresponding RTs to investigate the influence of error rates on parameter identifiability. The corresponding code and simulated data are available in the Supplementary Materials at https://osf.io/d389x/. The first study simulated a simple design where subjects respond to two different stimuli. The second and third studies added manipulations of stimulus difficulty and speed-vs-accuracy instructions, respectively. In each study, we examined three versions of the DDM and LBA that differed in the number of free parameters and range of data-generating values. As the conclusions were largely congruent, here we focus on the versions that resemble the models most commonly used in applications, producing errors that are slower than correct responses (see section Data Generation). We discuss the other two versions, where errors are faster than/equally fast as correct responses in the Discussion, and present details in the Supplementary Materials.

Parameter identifiability is typically investigated through parameter-recovery studies (Heathcote et al., 2015). First, data are generated from the model with a known set of parameters. Second, the same model is fit to the generated data to estimate its parameters. Third, the resulting parameter estimates are compared to the “true” data-generating parameters. Identifiability is implied if the estimates are close to the true values.

### Data generation

In Study 1, we generated synthetic datasets for seven different error rates (0, 1, 5, 15, 25, 35, and 50%), and in Study 2 and 3 for three different error rates (5, 25, and 45%). In Study 2, each dataset featured three conditions (easy, medium, and difficult) with equal trial numbers. For each error rate scenario, the easy condition had 2.5% less and the difficult 2.5% more errors than the target error rate (e.g., for a target error rate of 5%, the easy condition had 2.5%, the medium 5%, and the difficult 7.5% errors). In Study 3, each dataset featured two conditions (speed vs. accuracy instructions) with equal trial numbers, where the accuracy condition had 2.5% less and the speed condition 2.5% more errors than the target error rate. After evaluating the results of these simulations, we additionally generated datasets with 7.5, 10, and 12.5% errors for Study 1 to gain further insight into parameter identifiability within this critical region, which is commonly observed in experiments.

In Studies 1 and 2, we explored situations with 150 and 1200 trials per dataset; in Study 3, we used 200 and 1200 trials per dataset (the difference is due to the number of design cells over which trials were equally distributed). In the Supplementary Materials, we also report results for 450 and 750 trials for Studies 1 and 2, and 500 and 800 trials for Study 3. For each model, error rate, and trial number, 100 datasets were generated.

For the DDM, the diffusion coefficient was set to $s=1$, as is conventional, and non-decision time variability was fixed to $s_{t_0}=0$ as this parameter has no impact on error rates. For the LBA, we expressed decision thresholds as $B = b - A$ and followed the $s_{v_\text {false}} = 1$ convention for scaling.

All other data-generating parameter values were sampled from empirically observed distributions of parameter estimates. Kuhne (2024) used a bottom-up model selection approach (as described in Donkin et al., 2011) to obtain separate maximum likelihood estimates for each participant (Myung, 2003) for the DDM and LBA from 17 published experiments reporting RT and accuracy data in perceptual decision making, random dot motion, lexical decision, and various cognitive-conflict tasks. Their procedure started by fitting the most simple model (in terms of the number of parameters) to the data and then used the resulting parameter estimates as starting values to fit more complex models. Then, the best fitting model was chosen using the AIC (Akaike, 1974) and BIC (Schwarz, 1978). If the AIC and BIC selected different models, the AIC-selected model was chosen. In total, Kuhne (2024) estimated 580 parameter sets for the DDM and 765 for the LBA. The empirical distributions of parameter estimates were then transformed into a normal shape and fit with truncated multivariate normal distributions. Table 1 shows the correlation matrices of the truncated multivariate normal distributions for the DDM and LBA, respectively. These are between-subject correlations reflecting individual differences among estimated parameters within each model. We used these truncated multivariate normal distributions to sample the data-generating values for our simulations while taking the empirically observed between-subject correlation structure of the parameters into account.

**Table 1:** Correlation matrices of the multivariate normal distributions used for sampling the data-generating parameter values

	DDM						LBA				
	*a*	*v*	$s_v$	*z*	$s_z$		*A*	$t_0$	*B*	$s_{v_\text {true}}$	$v_{\text {true}}$
*v*	0.09					$t_0$	0.59				
$s_v$	0.49	0.67				*B*	-0.21	-0.50			
*z*	0.01	0.01	0.01			$s_{v_\text {true}}$	0.61	0.43	0.29		
$s_z^{'}$	0.01	0.41	0.44	0.00		$v_{\text {true}}$	0.54	0.33	0.30	0.87	
$t_0$	0.23	-0.03	-0.05	0.00	-0.15	$v_{\text {false}}$	0.38	0.09	0.26	0.50	0.49

The parameters from Kuhne (2024) were transformed to a normal shape prior to estimation of the correlation matrices. DDM = diffusion decision model; LBA = linear ballistic accumulator

As the error rate is determined by the data-generating parameters, we repeatedly generated datasets with different parameter samples until the following criteria were met:

1. For Study 1, the empirical error rate was within a margin of 1% around the target error rate (e.g., in the 5%-error scenario, datasets included between 4% and 6% errors). For Studies 2 and 3, the summed difference between empirical and target across conditions was within a margin of 2%.
2. To produce datasets with slower error than correct responses, the constraints $s_v > s_z$ (DDM) and $s_{v_\text {true}} > s_{v_\text {false}}$ (LBA) had to hold.
3. The RT distributions (in seconds) fulfilled the following criteria (adapted from Evans, 2020): $0.4 \le \text {Mean(RT)} \le 2.5$; $0.4 \le \text {Median(RT)} \le 2.5$; $0.1 \le \text {IQR(RT)} \le 2$^2^; $1.5 \le \text {Maximum(RT)}$; $0.5 \ge \text {Minimum(RT)}$.

These inclusion criteria ensured that the generated RT distributions were approximately in line with those observed in typical empirical studies. Datasets that failed to meet all three criteria were discarded, and replaced with a new one, with the maximum number of replacements set to 100, 000, after which no new dataset was returned.

For the 50%-error scenario in Study 1, no datasets generated by the DDM met all three criteria, most likely because parameters leading to such a high error rate could not be sampled from the empirical distribution. Specifically, it was impossible to sample drift rates sufficiently close to zero to obtain chance performance. In many applications, participants with near-chance performance are excluded because they are assumed to disregard the task instructions (Evans et al., 2017). However, it is theoretically interesting to explore the 50%-error scenario, so instead of sampling drift rates from the empirical distribution, we used a uniform distribution bounded by 0.005 and 0.05 to obtain sufficiently small drift rates to generate the targeted error rate of 50%.

In Studies 2 and 3, we modified the data generation procedure: We computed the $25^{th}$ and $75^{th}$ quantiles of the data-generating drift rates for the different error rates from Study 1 and matched them to the target error rates in Studies 2 and 3 (e.g., quantiles for drift rates in the 5% error condition in Study 1 would be matched to the 5% error conditions in Studies 2 and 3). New drift rates were sampled from a uniform distribution bounded by the two quantiles for the conditions in Studies 2 and 3. The other parameters were generated using the truncated multivariate normal distribution conditional on the new drift rates. In Study 3, parameters were first sampled for the accuracy condition (these were conditional only on the new drift rates). For the speed condition, we sampled a second decision threshold parameter conditional on the other newly generated model parameters (these were conditional on all parameters besides the threshold in the accuracy condition, including the new drift rates). This modification ensured ordinal consistency between the data-generating parameter values across conditions (e.g., lower drift rates in the difficult vs. easy condition).

Some of the DDM datasets, even though they met our inclusion criteria, were generated from parameters that were larger than typically observed in empirical studies. To ensure that the data-generating values align well with the literature at large, we removed datasets generated with $a > 3$ or $v > 5$ (Study 1: $20.8\%$; Study 2: $26.7\%$; Study 3: $35.9\%$). These thresholds were based on visual inspection of empirically observed parameter distributions reported by Tran et al. (2021). Removing these datasets did not change the conclusions of our simulations. In the Supplementary Materials, we show results that include all datasets and results that include only the excluded datasets.

For data generation, we used the Dynamic Model of Choice software (DMC; version: 210114; Heathcote et al., 2019) which relies on the R packages rtdists (version: 0.11-2; Singmann et al., 2020), which in turn uses parts of the fast-dm software (Voss & Voss, 2007). The simulations and analyses were run using R (version: 3.6.3; R Core Team, 2020).

### Parameter estimation

Response boundaries in the DDM and accumulators in the LBA were mapped to correct vs. incorrect responses. In Study 2, separate DDM *v* and LBA $v_{\text {true}}$ and $v_{\text {false}}$ parameters were estimated for the three difficulty conditions; in Study 3, separate DDM *a* and LBA *B* parameters were estimated for the speed-vs-accuracy conditions.

For each dataset, the joint posterior distributions of the model parameters were estimated with Bayesian differential-evolution Markov chain Monte Carlo (MCMC) sampling (Turner et al., 2013), as implemented in DMC. Specifically, parameters were estimated using the RUN.dmc function which repeatedly removes and adds samples to the MCMC chains until they converge to their stationary distribution (Heathcote et al., 2019). We ran 3*k* MCMC chains, where *k* is the number of free parameters in the model. Each chain was initialized with 120 samples and samples were added and removed in steps of 40 until (a) multivariate $\hat{R}$ and $\hat{R}$ for all parameters were below 1.1 (Brooks & Gelman, 1998); and (b) effective sample size (computed using the effectiveSize function from the coda R package; Plummer et al. , Plummer et al. ) for every parameter was at least 1000. These criteria ensured that the MCMC chains were well mixed and enough samples were available to robustly estimate summary statistics of the posterior distributions. We visually inspected random samples of chains to confirm convergence.

For the DDM, prior distributions were based on Tran et al. (2021), who provided an overview of DDM parameter estimates based on a systematic literature review. For the LBA, priors were adapted from Evans (2020). As both sets of prior distributions were relatively informative (i.e., had a narrow spread), we increased their variances to reduce their influence on the posterior distributions (see Supplementary Materials for details).

### Assessing parameter-recovery performance

We used the median of the marginal posterior distributions as point estimates for the parameters. We compared these point estimates to the true data-generating values using scatterplots, Pearson correlations, and the root mean squared deviation $RMSD = \sqrt{\frac{1}{n}\sum (x_{true} - x_{est})^2}$. RMSD can be compared across the various error-rate scenarios but not across parameters or models due to scale differences, whereas Pearson correlations can be compared across both. To assess the uncertainty of the results, we also calculated the 95% interval (i.e., $2.5^{th}$ and $97.5^{th}$ quantiles) of the absolute deviations between the true and estimated parameter values $abs(x_{true} - x_{est})$. To investigate parameter trade-offs, we computed pairwise Pearson correlations between MCMC samples comprising the marginal posterior distributions. To account for conditional dependencies, we also calculated *partial* pairwise correlations between the marginals. Importantly, the correlations encapsulated in the posterior distributions reflect the mathematical structure of the model and are defined within participants, whereas the correlations between data-generating parameter values (Table 1) reflect individual differences and are defined between participants. The two types of correlations therefore cannot be compared.

For Study 1, we focus on the core DDM and LBA parameters, and present results for the DDM $s_v$ and $s_z$ parameters and the LBA *A* parameter in the Supplementary Materials. For Studies 2 and 3, results related to DDM *z* and DDM and LBA $t_0$ are also relegated to the Supplementary Materials.

## Results

### Parameter-recovery performance

#### Study 1: stimulus manipulation

**Fig. 2:**
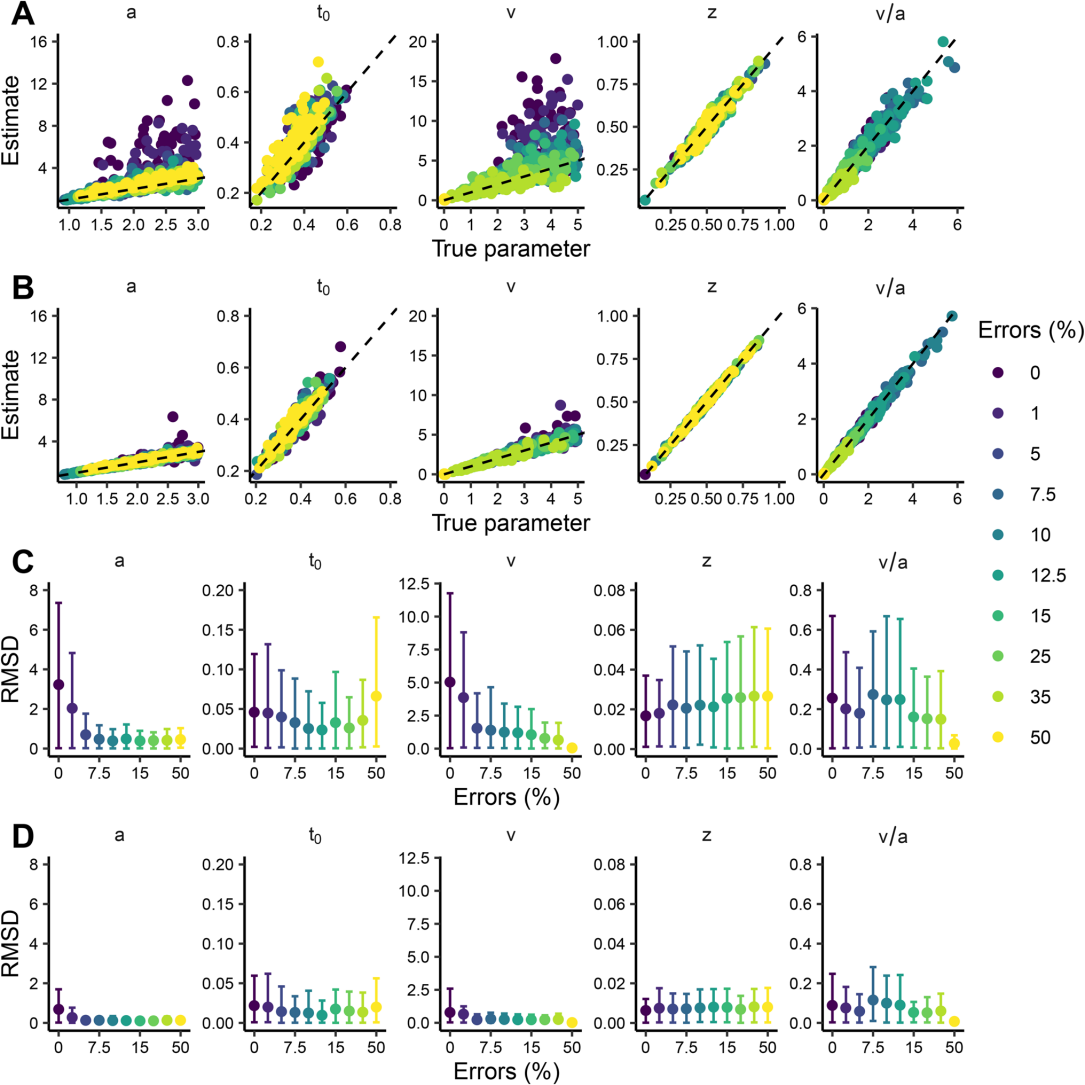
Parameter recovery for the diffusion decision model. **A**, **B**: Bivariate distributions of true values and posterior medians for the diffusion decision model for different error rates (*colors*). Each *point* represents a dataset of 150 (**A**) or 1200 (**B**) trials; the *dashed line* represents perfect recovery. **C**, **D**: Root mean squared deviation (RMSD) between true values and posterior medians for 150 and 1200 trials, respectively, for different error rates. *Error bars* indicate the $2.5^{th}$ and $97.5^{th}$ quantiles of the absolute deviations. Note that the *x*-axis in C and D is not linear and has a discrete scale

Figure 2A and B show scatterplots of true vs. estimated DDM parameters across 100 synthetic datasets for different error rates. For 150 trials per dataset (Fig. 2A), estimates of *a* and *v* increasingly overestimate the true values as error rates decrease. The overestimation is still present for 1200 trials (Fig. 2B) with 0% and 1% errors, but it is substantially smaller than for 150 trials. The overestimation is reflected in weaker correlations between true and estimated parameters as error rates decrease (Table 2). These results are supported by Fig. 2C and D, where RMSDs of *a* and *v* are generally higher for lower error rates. RMSDs decline steeply from 0% to 5% (for both *a* and *v*) and more gradually for higher error rates. For $t_0$ and *z*, estimates align well with true parameter values even at low error rates, with RMSDs being relatively stable across the different error scenarios.

**Fig. 3:**
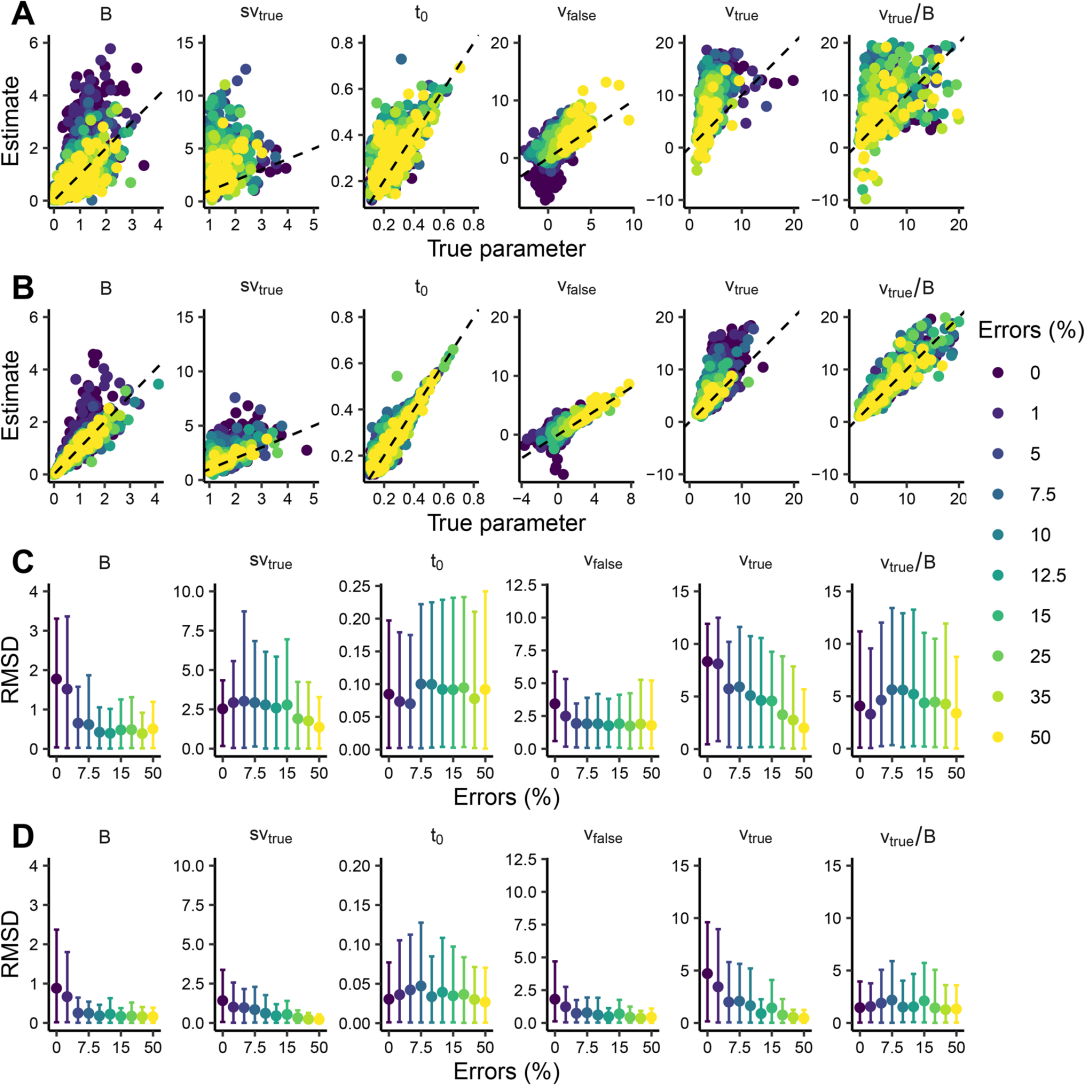
Parameter recovery for the linear ballistic accumulator. **A**, **B**: Bivariate distributions of true values and posterior medians for the linear ballistic accumulator for different error rates (*colors*). Each *point* represents a dataset of 150 (**A**) or 1200 (**B**) trials; the *dashed line* represents perfect recovery. **C**, **D**: Root mean squared deviation (RMSD) between true values and posterior medians for 150 and 1200 trials, respectively, for different error rates. *Error bars* indicate the $2.5^{th}$ and $97.5^{th}$ quantiles of the absolute deviations. Note that the *x*-axis in C and D is not linear and has a discrete scale

Figure 3A and B show scatterplots of true vs. estimated LBA parameters across error rates. Estimates of *B*, $s_{v_\text {true}}$, $v_{\text {false}}$, and $v_{\text {true}}$ are biased upwards for decreasing error rates for 150 trials. The bias largely disappears above 5% errors for 1200 trials. The bias is also evident in correlations between true and estimated parameters in Table 2, where correlations are weaker for lower error rates. Notably, correlations between true and estimated LBA parameters are lower overall than for the DDM, both with 150 and 1200 trials. Figure 3C and D show that RMSDs increase with decreasing error rates, except for $t_0$.

**Table 2:** Pearson correlations between true and estimated parameters

	DDM					LBA					
Errors ($\%$)	*a*	$t_{0}$	*v*	*z*	*v*/*a*	*B*	$s_{v_{\text {true}}}$	$t_0$	$v_{\text {false}}$	$v_{\text {true}}$	$v_{\text {true}}/B$
150 trials											
0.0	0.47	0.83	0.29	0.96	0.93	0.41	0.13	0.64	0.08	0.19	0.34
1.0	0.55	0.77	0.50	0.95	0.94	0.47	-0.07	0.72	0.22	0.01	0.58
5.0	0.72	0.89	0.76	0.96	0.96	0.70	0.31	0.81	0.38	0.28	0.49
7.5	0.83	0.90	0.69	0.97	0.97	0.55	0.21	0.53	0.58	0.42	0.42
10.0	0.91	0.93	0.77	0.96	0.97	0.71	0.18	0.64	0.66	0.52	0.50
12.5	0.88	0.94	0.75	0.97	0.97	0.75	0.37	0.69	0.67	0.57	0.37
15.0	0.85	0.88	0.82	0.93	0.94	0.71	0.16	0.66	0.64	0.47	0.65
25.0	0.84	0.93	0.84	0.97	0.91	0.62	0.43	0.61	0.57	0.54	0.56
35.0	0.88	0.86	0.80	0.96	0.91	0.79	0.28	0.70	0.72	0.65	0.44
50.0	0.89	0.81	-0.09	0.95	0.33	0.64	0.62	0.66	0.74	0.72	0.62
1200 trials											
0.0	0.60	0.95	0.85	1.00	0.99	0.61	0.55	0.92	0.46	0.59	0.90
1.0	0.81	0.96	0.81	0.99	0.99	0.72	0.51	0.90	0.56	0.61	0.88
5.0	0.96	0.97	0.97	0.99	0.99	0.89	0.62	0.89	0.68	0.73	0.90
7.5	0.98	0.98	0.95	1.00	1.00	0.87	0.73	0.90	0.79	0.77	0.89
10.0	0.98	0.97	0.96	1.00	1.00	0.96	0.62	0.94	0.76	0.72	0.93
12.5	0.98	0.99	0.96	0.99	0.99	0.94	0.81	0.91	0.86	0.80	0.90
15.0	0.98	0.96	0.97	0.99	0.99	0.95	0.59	0.94	0.81	0.77	0.87
25.0	0.98	0.97	0.97	1.00	0.99	0.95	0.80	0.94	0.91	0.85	0.94
35.0	0.97	0.98	0.96	1.00	0.97	0.95	0.84	0.95	0.92	0.90	0.92
50.0	0.98	0.96	0.31	1.00	0.54	0.95	0.91	0.97	0.94	0.93	0.94

Correlations for DDM *v* are low at 50% errors due to low variance in the true parameters. DDM = diffusion decision model; LBA = linear ballistic accumulator

#### Studies 2 and 3: difficulty and speed-vs-accuracy manipulation

**Fig. 4:**
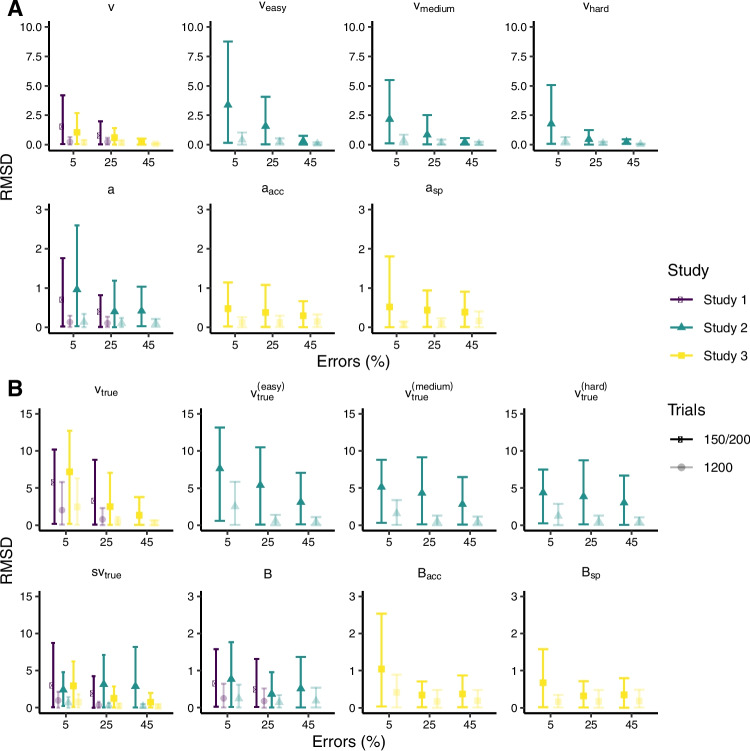
Parameter recovery for the diffusion decision model and the linear ballistic accumulator with a difficulty and speed–accuracy manipulation. Root mean squared deviation (RMSD) between true values and posterior medians for the diffusion decision model (**A**) and the linear ballistic accumulator (**B**) for different error rates (*x*-axis), studies (*color* and *point shape*) and trial numbers (*opacity*). *Error bars* indicate the $2.5^{th}$ and $97.5^{th}$ quantiles of the absolute deviations. Study 2 has separate drift rates for difficulty conditions (easy, medium, hard) and Study 3 separate decision thresholds for speed (sp) and accuracy (acc) conditions. Studies 1 and 2 have datasets with 150 trials and Study 3 has datasets with 200 trials. Note that the *x*-axis is not linear and has a discrete scale

Figure 4A shows RMSDs for the DDM with a simulated difficulty (Study 2) and speed-vs-accuracy manipulation (Study 3) for different error rates and trial numbers. As in Study 1, RMSDs of *a* and *v* are higher for decreasing error rates and for 150 (Study 2) and 200 (Study 3) compared to 1200 trials. RMSDs are also higher for drift rates estimated for conditions with lower difficulty (as these have a lower error rate) and similar to RMSDs for drift rates in Study 1 with a comparable error rate of 5%. RMSDs for decision thresholds are similar in accuracy and speed conditions. Interestingly, RMSDs for decision thresholds and drift rates are overall lower for the speed-vs-accuracy manipulation than for the difficulty manipulation. They are also lower than RMSDs for decision thresholds in Study 1 with a comparable error rate of 5%. This is true even for $a_{acc}$ in the accuracy condition which was estimated with a lower error rate and smaller trial number than *a* in the difficulty manipulation.

Figure 4B shows RMSDs for the LBA with a simulated difficulty and speed-vs-accuracy manipulation. Again, RMSDs are generally higher for decreasing error rates and for 150 compared to 1200 trials, as well as for parameters in conditions with lower error rates (e.g., $v^{(\text {easy})}_{\text {true}}$ or $B_{\text {acc}}$). In contrast to the DDM, only $s_{v_\text {true}}$ appears to have lower RMSDs for the speed-vs-accuracy manipulation with 200 trials and 25% and 45% errors. Other parameters show no systematic differences between the manipulations. In the Supplementary Materials, we also show parameter estimates from Studies 2 and 3.

### Pairwise within-subject correlations

**Fig. 5:**
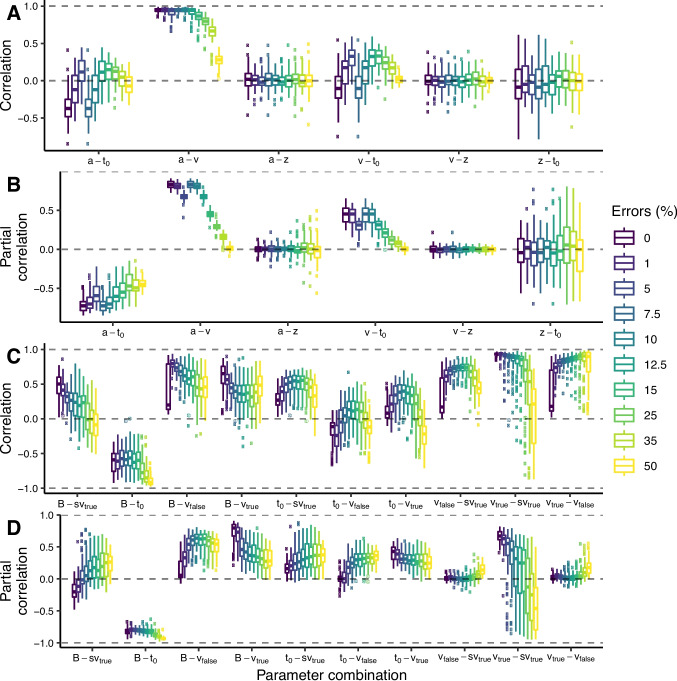
Pairwise within-subject correlations between posterior distributions for each model. **A**, **C**: Distribution of the Pearson correlation between the marginal posterior distributions across the 100 synthetic datasets, each with 150 trials, for the diffusion decision model (DDM) and the linear ballistic accumulator (LBA), respectively. **B**, **D**: Distribution of partial correlations for the DDM and the LBA, respectively. *Boxplot* hinges show the $25^{th}$ and $75^{th}$ quantiles; *whiskers* point to 1.5 times the interquartile range beyond the hinges; correlations beyond the whiskers are shown as outliers

Figure 5A shows distributions of pairwise Pearson correlations between DDM parameters across the 100 synthetic datasets, each with 150 trials, for different error rates. Correlations for 1200 trials, which closely mirror the results in Fig. 5, are reported in the Supplementary Materials. Correlations between $a-v$ are very close to 1 at lower error rates and start decreasing at 15% errors and above. The near-perfect correlation between $a-v$ at low error rates indicates a trade-off, where simultaneously increasing *a* and *v* leads to (almost) identical predictions.

Trade-offs between other DDM parameters can be more easily observed when accounting for conditional dependencies, as shown with the partial correlations in Fig. 5B. Between $a-t_0$, partial correlations tend to become more negative with decreasing error rates, suggesting a trade-off where decreasing *a* and increasing $t_0$ leads to (almost) identical predictions. Partial correlations between $a-v$ and also $v-t_0$ decrease from close to 1 in the 0% scenario to 0 in the 50% scenario, supporting our earlier conclusion based on Pearson correlations and again suggesting trade-offs between these parameters.

Figure 5C shows correlations between LBA parameters for different error rates. Correlations are typically more extreme (i.e., closer to 1 or -1) than in the DDM. We observe correlations close to 1 at low error rates between $B-s_{v_\text {true}}$, $B-v_{\text {false}}$, $B-v_{\text {true}}$, $v_{\text {false}}-s_{v_\text {true}}$, and $v_{\text {true}}-s_{v_\text {true}}$. Correlations between $v_{\text {true}}-v_{\text {false}}$ are close to 1 regardless of error rate. Interestingly, correlations between $B-t_0$ seem to approach $-1$ as error rates increase. These results indicate strong and frequent parameter trade-offs, especially in the presence of near-chance or near-perfect performance.

As shown in Fig. 5D, pairwise partial correlations in the LBA follow a qualitatively different pattern than Pearson correlations. Correlations generally become less extreme when we account for conditional dependencies, and some even switch signs (e.g., $B - s_{v_\text {true}}$). The patterns for $B-v_{\text {true}}$ and $v_{\text {true}}-s_{v_\text {true}}$ remain largely unchanged, i.e., strong positive correlations for low error rates that decrease, and even reverse for $v_{\text {true}} - s_{v_\text {true}}$, as error rates increase. The pattern for $B - t_0$ also remains similar in so far that correlations approach $-1$ as error rates increase, but as opposed to Pearson correlations, the strength of partial correlations is only weakly influenced by error rates.

Lastly, we investigated whether there are *combinations* of parameters that can be adequately recovered even if their constituents are poorly estimated due to strong trade-offs (as has been done for the LBA by Evans (2020), and for conflict diffusion models by White et al. (2018)). For the DDM, we examined the ratio of *v*/*a* and for the LBA $v_{\text {true}}/B$ because they showed similarly high Pearson and partial correlations indicating strong trade-offs independent of other model parameters. As shown in the most right panels in Figs. 2A-D and 3A-D as well as Table 2, ratios are substantially better recovered than individual parameters at low error rates, except for the LBA with 150 trials.

## Discussion

In three simulation studies, we investigated how error rates influence the identifiability of parameters in two prominent evidence-accumulation models, the diffusion decision model (DDM) and the linear ballistic accumulator (LBA). We also examined relationships between parameter estimates within each model, searching for parameter trade-offs that might contribute to estimation difficulties at low error rates.

We observed increasing bias as error rates decreased for *v* and *a* in the DDM and $v_{\text {true}}$ and *B* in the LBA. The biases were stronger for small than large trial numbers (150 vs. 1200). In Studies 2 and 3, we showed that adding a speed-vs-accuracy manipulation with selective influence on the parameters reduced the bias at low error rates for the DDM but not for the LBA. The difficulty manipulation did not reduce the bias in either model. Overall, the bias at low error rates was less pronounced for the DDM than the LBA, especially for small trial numbers.

Pairwise Pearson and partial correlations between marginal posterior distributions revealed strong positive relationships between *v* and *a* in the DDM, and *B* and $v_{\text {true}}$ in the LBA at low error rates. These high correlations occur because, at low error rates, the models are almost exclusively constrained by RTs. Different pairs of $v-a$ and $B-v_{\text {true}}$ can lead to similar RT distributions causing trade-offs and hence high parameter correlations. The fact that these within-person posterior correlations did not differ systematically between low and high trial numbers indicates that parameter trade-offs at low error rates become less problematic as the number of trials increases. Therefore, it is likely that the absolute number of error trials is what matters most for parameter identifiability.

We investigated parameter trade-offs further by looking at estimates of ratios of parameter pairs. For DDM *v* and *a*, parameter ratios were recovered substantially better for small trial numbers and excellently for large trial numbers across all error rates. For LBA $v_{\text {true}}$ and *B*, this was only evident for large trial numbers. For small trial numbers, $v_{\text {true}}/B$ was less well recovered than the individual parameters. For 150 trials and low error rates, the data might not provide enough information to estimate LBA parameters or their ratios with sufficient accuracy, irrespective of correlations between parameter posteriors. Moreover, we found strong negative dependencies between DDM *a* and $t_0$ as well as LBA *B* and $t_0$. However, the dependencies did not compromise parameter recovery across error rates since $t_0$ only affects the leading edge of RT distributions and not accuracy.

As detailed in the Supplementary Materials, for simpler models with no between-trial parameter variability (e.g., DDM $s_v= s_z=0$ and LBA $A=0$), identifiability was generally better. In parameter regions producing faster error than correct responses, identifiability was comparable to or slightly worse than in slow-error regions. In Study 2, drift rates decreased with increasing difficulty, which is consistent with Ratcliff (2014), but were biased at low error rates (see also Fig. 4). Thus, our results show that ordinally consistent yet poorly identified estimates can be obtained at low error rates when drift rates are estimated separately for difficulty levels. In Study 3, separate decision threshold parameters for response caution levels were well identified at low error rates when drift rate estimates were shared across levels.

To strengthen our conclusion that parameter identifiability was compromised by *within*-subject parameter trade-offs and were not the result of *between*-subject correlations between the parameters in each model, we also looked at correlations between data-generating parameters in datasets that satisfied our inclusion criteria. As shown in the Supplementary Materials, these correlations are low and do not systematically vary with the error rate for most parameters. Exceptions are $v_{\text {true}}$ and $v_{\text {false}}$ in the LBA. However, the correlations between these two parameters are low at low error rates and the opposite of what would be required to explain our results.

Lastly, we conducted a post hoc analysis of how well group differences could be identified at low error rates (see Supplementary Materials). For Studies 2 and 3, we created a between-group manipulation by treating datasets with slow and fast errors as independent experimental groups. For each group, we calculated the mean of the posterior medians for each parameter of each model. Then, we compared the group difference between estimated means to the difference between true means. For both studies, the analysis showed a bias where the mean group difference in drift rates was largely overestimated at low error rates. Despite being a crude approach to recovering group differences, it indicates that low error rates *can* interact with experimental manipulations biasing estimates of group differences in parameters.

Why are the DDM parameters poorly identified at extremely low error rates? To produce low error rates, the drift rate must be high relative to the distance from the starting point of evidence accumulation to the response threshold, so that the lower, incorrect, boundary is (almost) never hit. Similarly, in the LBA, the matching accumulator tends to (almost) always win if $v_{\text {true}}$ is high relative to *B*. In both cases, the parameter pairs can trade off to produce almost identical model predictions because their ratio influences the predictions instead of their individual values. The trade-offs lead to high correlations between the parameter estimates and poor parameter identifiability.

Why is the LBA more prone to these effects than the DDM? This is likely because the DDM assumes a simpler one-dimensional process driven by only the difference between the evidence for each response, whereas the LBA (and other racing accumulator models) are also sensitive to the magnitudes of each type of evidence (e.g., Miletić et al., 2021). When error rates are low, there is little information available to differentiate difference and magnitude effects, and so identifiability suffers.

Our results are in line with those of Ratcliff and Tuerlinckx (2002) who also observed a larger variation of estimates around true parameters for higher values of the DDM *a* and *v*, which produce lower error rates. Similarly, Lerche et al. (2017) found that more trials are required to reliably estimate *a* and *v* when fewer than 4% of the responses reached the upper or lower decision boundary, respectively. For the LBA, we confirm findings by Heathcote et al. (2019) that lower error rates impair the recovery of LBA parameters and provide a more comprehensive picture of the relationship between error rates and parameter identifiability. Our results can also explain the seemingly diverging results of Heathcote et al. (2019); Evans (2020); Palestro et al. (2018). Evans (2020) reported poor identifiability for high values of LBA $v_{\text {true}}$ and *B* and suspected a trade-off between these parameters, leading to the recommendation to use a constrained version of the LBA where $v_{\text {true}}/B$ is estimated. However, high $v_{\text {true}}$ and *B* parameters produce data with low error rates, which we showed causes poor identifiability. In contrast, Palestro et al. (2018) generated data with $\sim 30$% errors and reported good recovery, similar to Heathcote et al. (2019) with 25% errors. Thus, instead of Evan’s conclusion that the standard LBA is an inherently flawed model that should generally not be used, our results indicate that it has an identifiability issue at low error rates, as does the DDM but is applicable in situations where error rates are higher.

Based on our findings, we recommend future studies using the DDM and LBA to aim for choice tasks resulting in error rates between 15% and 35%. For small trial numbers (e.g., 150), error rates below 15% could compromise parameter identifiability for both models. For very small trial numbers ($< 150$), although not explicitly investigated here, we expect identifiability at low error rates to be even worse. For large trial numbers, we recommend error rates of at least 5%, although for the DDM 2% may still be sufficient. Generally, higher error rates are more important for the LBA than for the DDM. We do not advise going much beyond 35% error rates as near-chance-level performance compromises the interpretability of the parameters. If accuracy in a task is low, our results suggest that manipulating response caution improves the identifiability of the DDM parameters more than manipulating task difficulty. We note that avoiding accuracy ceiling effects is also important for traditional analyses of choice data; ceiling effects reduce the variability in the data, which can bias correlations and decrease power.

Our parameter-recovery study relied exclusively on differential-evolution Markov chain Monte Carlo (DE-MCMC) sampling for parameter estimation. Other estimation methods, for instance, those based on quantiles (Heathcote, 2004), may recover parameters differently across error rates. However, because DE-MCMC showed robust estimation of highly correlated parameters (Turner et al., 2013), it likely performs better than methods that are not equipped to deal with sloppiness.

### Future directions

Future studies can build on ours in multiple ways. Our systematic methodology might be used to investigate other EAMs, such as the increasingly popular racing diffusion model (Tillman et al., 2020), which combines elements of the DDM and the LBA (see Castro et al., 2019, for a parameter recovery study focused on their specific design). Similarly, it seems likely to be worthwhile to investigate the effect of errors in other models with potential identifiability issues (e.g., White et al., 2018).

Our study based estimation on single-subject data, although hierarchical estimation is common in the contemporary EAM literature (Heathcote et al., 2019; Wiecki et al., 2013). Future studies could examine if shrinkage effects associated with hierarchical estimation (see Boehm et al., 2018) can be used to improve parameter identifiability at low error rates.

Lastly, our results can be treated as an upper bound for identifiability under ideal conditions. It is important to assess whether our simulation results generalize to empirical data. This is challenging because the data-generating parameter values in empirical data are unknown. The comparison of empirically obtained and simulated pairwise correlations between marginal posteriors may provide a solution. If empirical correlations align with simulated correlations, identifiability patterns for empirical and simulated data will also likely align.

### Supplementary Materials

https://osf.io/d389x/

## Data Availability

The code used for the current study is available in the Open Science Framework repository, https://osf.io/d389x/.

## Footnotes

1 Models with non-identified parameters can still be theoretically informative and used for prediction or in model comparisons (Evans & Wagenmakers, 2019).

2 IQR = interquartile range

## Availability of Data and Materials

The datasets generated during and/or analyzed during the current study are available in the Open Science Framework repository, https://osf.io/d389x/, and figshare repository, https://figshare.com/account/home#/projects/181213. None of the simulation studies were preregistered.
